# Concomitant Guillain-Barre syndrome with COVID-19: a case report

**DOI:** 10.1186/s12883-021-02162-3

**Published:** 2021-03-23

**Authors:** Nuvia Mackenzie, Eva Lopez-Coronel, Alberto Dau, Dieb Maloof, Salvador Mattar, Jesus Tapia Garcia, Briyis Fontecha, Cristina M. Lanata, Hernan Felipe Guillen-Burgos

**Affiliations:** 1 Center for Clinical and Translational Research, La Misericordia Clínica Internacional, Barranquilla, Colombia; 2 Rosalind Russell/Ephraim P Engleman Rheumatology Research Center, University of California, San Francisco, California USA; 3 Universidad Simón Bolívar, Facultad de Ciencias de la Salud, Barranquilla, Colombia

**Keywords:** COVID-19, SARS-CoV-2, Guillain-Barre syndrome, Case report

## Abstract

**Background:**

During the Coronavirus disease 2019 (COVID-19) pandemic, different neurological manifestations have been observed. However, only a few cases of Guillain-Barre syndrome (GBS) and COVID-19 have been reported. Therefore, the aim of this study is to investigate a case of concomitant GBS with COVID-19 in Colombia.

**Case presentation:**

A 39-year-old woman was admitted to a teaching hospital in Barranquilla, Colombia with a history of progressive general weakness with lower limb dominance. Previous symptoms such as ageusia, anosmia and intense headache were reported. Upon admission, facial diplegia, quadriparesis with lower extremity predominance and Medical Research Council muscular strength of 2/5 in the lower limbs and 4/5 in the upper limbs were reported. During clinical evolution, due to general areflexia, hypertensive emergency and progressive diaphragmatic weakness, the patient was admitted to an intensive care unit. The cerebrospinal fluid analysis showed protein-cytological dissociation and the GBS diagnosis was confirmed via a nerve conduction and electromyography test. With regard to the symptoms before hospitalisation, SARS-CoV-2 diagnostic testing was performed with positive results in the second test. The patient was managed with supportive care and was discharged after 20 days of hospitalization with clinical improvement.

**Conclusions:**

Only a few cases of COVID-19 with GBS have been reported. Different subtypes have been previously identified, such as Miller-Fisher syndrome and dysautonomic GBS with SARS-CoV-2 infection. This study investigated the first confirmed case of COVID-19 with concomitant GBS in Colombia. In patients with GBS, several viral and bacterial pathogens have been found in case-control studies but the factors that induce the immune-mediated destruction of the nerve tissues have not been determined. Further studies are needed to determine the possible association between COVID-19 exposure and GBS.

## Background

A novel coronavirus emerged in Wuhan, China in late December 2019 generating a pandemic disease named coronavirus disease 2019 (COVID-19) caused by the SARS-CoV-2 virus [1]. The rapid spread of the virus from China to Europe, the United States and currently Latin American countries has caused several deaths and cumulative cases in countries with overwhelmed sanitary systems, such as Italy, Spain, the United States and several regions in South America [2]. Moreover, the United States and Brazil were the first and second hotspots the disease with more cases and deaths reported, respectively [3]. In Colombia to date, more than 2.161.462 cumulative cases and more than 56.290 deaths [4] with cluster regions, such as Bogota, D. C, and Barranquilla have been reported. Furthermore, this brief report investigates a case of COVID-19 with the concomitant Guillain Barre syndrome (GBS) variant from the Hospital La Misericordia Clínica Internacional in Barranquilla, Colombia.

## Case presentation

During mid-April 2020, a 39-year-old woman with previous medical history of hypertension controlled with medication, type 2 diabetes mellitus of recent diagnosis controlled with medication, and cholecystectomy (6 years ago) was admitted to a teaching hospital in Barranquilla, Colombia, and presented with symptoms of progressive generalised weakness for 6 days, predominantly involving the lower limbs. Twenty days prior to admission, she had an episode of illness consisting of ageusia, anosmia and intense headache for which she had multiple consultations without improvement of the symptoms. She also complained of intense myalgias and symmetric, ascendant and generalised weakness in the lower limbs, which gradually progressed to inability to walk for which she was referred to our hospital. Upon admission, the patient reported symptoms of headache, malaise, generalised myalgias, cough without expectoration and inability to walk. Her blood pressure on sitting was 167/88 mmHg, heart rate was regular (74 beats per min), respiratory rate was 18 breaths per min (bpm) and SaO_2_ was 95% on room air. Based on her neurological examination, she was found to have peripheral facial diplegia, generalised hyporeflexia, generalised quadriparesis with lower limb predominance, Medical Research Council (MRC) muscular strength of 2/5 in the lower limbs and 4/5 in the upper limbs. She also had left arm paraesthesia. No affectation was observed in the bulbar muscles. During the clinical evolution she had generalised flaccid areflexia. She was later shifted to the intensive care unit due to hypertensive emergency (189/112 mmHg), and progressive diaphragmatic weakness with 36 bpm, without breath sounds in auscultation, which could indicate dysautonomic symptoms. Table 1 shows the results of the initial laboratory tests indicating leukocytosis, neutrophilia (79.90%, with a neutrophil-lymphocyte ratio of 16.27), lactate dehydrogenase and elevated D-Dimer levels. Creatine phosphokinase (CPK) levels were normal. The cerebrospinal fluid analysis revealed protein cytological dissociation (298.3/0.05). Elevated cerebrospinal fluid (CSF) protein is a hallmark of GBS. The chest X-ray and chest CT scan (128-slice SOMATOM, Siemens, GE) were normal. A nerve conduction and electromyography test (Table 2) confirmed the clinical evaluation of the GBS diagnosis. The MRI showed an incipient degenerative cervical disc disease at the C5-C6 level, normal thoracic spine, a Pfirrmann IV degenerative lumbar disc disease with central protrusions and flavum ligamentum hypertrophy at levels L3-L4, L4-L5 and L5-S1, which are not related with the clinical signs and symptoms of the patient.

**Table 1:** Laboratory testing upon admission

Laboratory testing upon admission		
**Complete blood count**		**Reference range**
Total leucocyte count	12.57 (10^3^/mm^3^)	4–10.04 (10^3^/mm^3^)
Neutrophils	79.9%	34–71.1%
Lymphocytes	13%	19.3–51.7%
Monocytes	6.6%	4.7–12.6%
Eosinophils	0.4%	0.7–5.8%
Basophils	0.1%	0.1–1.2%
Haematocrit	35.6%	34.1–44.9%
Haemoglobin	11.2 g/dL	11.2–15.7 (gr/dL)
Platelets	232 (10^3^ uL)	150–450 (10^3^ uL)
**Electrolytes**		
Sodium	136 mmol/L	135–148 mmol/L
Potassium	4.57 mmol/L	3.3–5.1 mmol/L
Chloride	96.5 mmol/L	98–107 mmol/L
**Blood glucose levels**		
Glycemia	240 (mg/dL)	70–105 (mg/dL)
**Others**		
Lactate dehydrogenase	306 (U/L)	140–280 U/L
Ferritin	114.5 ng/mL	15–150 ng/mL
D-dimer	517 ng/mL	< 250 ng/mL
Serum creatinine	0.51 mg/dL	0.5–1.1 mg/dL
Erythrocyte sedimentation rate (ESR)	65	0–25 mm/hour

**Table 2:** Electromyography test

Nerve conduction study	Stimulation point	Record point	Distal latency (ms)	Amplitude (mV)	Velocity (m/s)
**Motor NCS**					
Right tibial nerve	Ankle-popliteal fossa	ABD halluc is brevis	14.1	3.2	19.9
Right common peroneal nerve	Ankle-head of fibula	EXT digitorum brevis	13.7	3.0	19.4
Left tibial nerve	Ankle-popliteal fossa	ABD halluc is brevis	14.7	3.4	19.7
Right median nerve	Wrist-elbow	ABD pollicis brevis	26.3	4.2	23.8
Left median nerve	Wrist-elbow	ABD pollicis brevis	24.2	4.5	23.4
**Sensitive NCS**					
Right sural nerve	Ankle	Calf	14.9	3.2	–

The patient was managed with supportive care; administered with enoxaparin (40 mg SC per day), losartan (50 mg BID), meperidine IV for muscle pain, hydroxychloroquine (400 mg twice first day and later on 200 mg BID per 10 days) and dexamethasone (8 mg every 8 h during 3 days); and received standard care. She did not require supplemental oxygen or ventilation, and no alterations were observed in the chest images. She also received plasmapheresis for 5 days, one session per day. After three sessions, the patient had neurological improvement, (MRC) muscular strength of 3/5 in the lower limbs, and improved respiratory rate (20 bpm). During the hospitalization, the patient underwent two nasal swab tests for SARS-CoV-2. The first RT-PCR test (Charité/Berlin Protocol) for SARS-CoV-2 was negative, whereas the second RT-PCR test (Charité/Berlin Protocol) for SARS-CoV-2 was positive. She was discharged after 20 days of hospitalisation with improvement of neurological status (MRC of 4/5 in the lower limbs) and respiratory symptoms.

## Discussion and conclusions

Neurological outcomes in patients with COVID-19 have been reported [5]. The neurological manifestations range from headache, dizziness, confusion, to in a few cases, more severe conditions, such as encephalopathy, acute disseminated encephalomyelitis and GBS, among others [6].

GBS is a neuroinflammatory disease with a global incidence of 1–2 per 100,000 person-years [7]. GBS is the most common cause of acute flaccid paralysis and its diagnosis includes patient history, neurological examination, electrophysiological test and CSF analysis [7]. Several viral and bacterial pathogens have been found in GBS patients in case-control studies, but the factors that induce the immune-mediated destruction of the nerve tissues have not been determined [8]. Moreover, the association between previous exposition to pathogens (e.g. cytomegalovirus, Epstein-Barr virus, *Mycoplasma pneumonia*, Zika virus, *Campylobacter jejuni*. and novel coronavirus SARS-CoV-2) and GBS outcome has been reported [9–13].

This study reports the first case of concomitant GBS with COVID-19 with a travel history from Wuhan [14], followed by other cases in countries with a high incidence of SARS-CoV-2, such as Italy [13], Iran [15] and Spain [16]. Other subtypes of GBS have been identified with SARS-CoV-2 infection such as Miller-Fisher syndrome (MLS) [17] and dysautonomic GBS [18]. Although a possible hypothesis of GBS caused by COVID-19 has been proposed, only fewer cases have been reported [19].Nevertheless, in patients with GBS and COVID-19, it not possible to identify SARS-CoV-2 virus via CSF analysis. A retrospective systematic screening of CSF samples from COVID-19 patients, showed negative findings [20].

We speculate that polyneuropathies, such as GBS, could be identified in COVID-19 patients, but more studies are needed to explore the neurological outcomes of these kinds of patients. It is not clear whether the infection preceded the GBS, or whether the infection is concomitant or considered a casualty in this group of patients. Because GBS is rare and the pandemic continues, the information contained in this report is very important for future research. More studies are needed to determine if COVID-19 has a direct association with GBS, such as its previously reported association with the Zika virus (ZIKV) [12]. During the last outbreak of the ZIKV in Colombia, the researchers reported virological evidence of ZIKV in patients with GBS in Colombia [12].

In summary, this study reported the first case of concomitant GBS with COVID-19 in Colombia, with favourable outcomes. As the pandemic continues, we would be able to determine whether this potential association could be attributed to the increase in GBS cases.

## Data Availability

All data are included in the electronic medical record of the patient. The datasets generated and/or analysed during the current study are not publicly available due on our policy statement of sharing clinical data only on request but are available from the corresponding author on reasonable request.

## Abbreviation

BID: Twice a day, *bis in die*
COVID-19: Coronavirus disease 2019
CPK: Creatine phosphokinase
CSF: Cerebrospinal serum fluid
GBS: Guillain-Barre syndrome
LDH: Lactate dehydrogenase
MRC: Medical Research Council
RT-PCR: Reverse transcription-polymerase chain reaction
SARS-CoV-2: Severe acute respiratory syndrome coronavirus 2
SC: Subcutaneous
ZIKV: Zika virus
